# Meta-analysis of longitudinal neurocognitive performance in people at clinical high-risk for psychosis

**DOI:** 10.1017/S0033291722001830

**Published:** 2022-08

**Authors:** Emily P. Hedges, Cheryl See, Shuqing Si, Philip McGuire, Hannah Dickson, Matthew J. Kempton

**Affiliations:** 1 Department of Psychosis Studies, Institute of Psychiatry, Psychology & Neuroscience, King's College London, London SE5 8AF, UK; 2 Department of Forensic and Neurodevelopmental Sciences, Institute of Psychiatry, Psychology & Neuroscience, King's College London, London SE5 8AF, UK

**Keywords:** CHR, cognition, processing speed, psychosis, UHR

## Abstract

Persons at clinical high-risk for psychosis (CHR) are characterised by specific neurocognitive deficits. However, the course of neurocognitive performance during the prodromal period and over the onset of psychosis remains unclear. The aim of this meta-analysis was to synthesise results from follow-up studies of CHR individuals to examine longitudinal changes in neurocognitive performance. Three electronic databases were systematically searched to identify articles published up to 31 December 2021. Thirteen studies met inclusion criteria. Study effect sizes (Hedges' *g*) were calculated and pooled for each neurocognitive task using random-effects meta-analyses. We examined whether changes in performance between baseline and follow-up assessments differed between: (1) CHR and healthy control (HC) individuals, and (2) CHR who did (CHR-T) and did not transition to psychosis (CHR-NT). Meta-analyses found that HC individuals had greater improvements in performance over time compared to CHR for letter fluency (*g* = −0.32, *p* = 0.029) and digit span (*g* = −0.30, *p* = 0.011) tasks. Second, there were differences in longitudinal performance of CHR-T and CHR-NT in trail making test A (TMT-A) (*g* = 0.24, *p* = 0.014) and symbol coding (*g* = −0.51, *p* = 0.011). Whilst CHR-NT improved in performance on both tasks, CHR-T improved to a lesser extent in TMT-A and had worsened performance in symbol coding over time. Together, neurocognitive performance generally improved in all groups at follow-up. Yet, evidence suggested that improvements were less pronounced for an overall CHR group, and specifically for CHR-T, in processing speed tasks which may be a relevant domain for interventions aimed to enhance neurocognition in CHR populations.

## Introduction

Robust deficits in neurocognition are evident in the early stages of psychosis development among people at clinical high-risk for psychosis (CHR) (Catalan et al., 2021; Hedges et al., 2022; Seidman et al., 2016). As these deficits are less pronounced than individuals with first-episode psychosis (FEP) compared to healthy control (HC) individuals (Sheffield, Karcher, & Barch, 2018), reviews indirectly comparing cross-sectional studies of FEP and CHR samples have suggested a potential neurocognitive decline prior to or over the transition to psychosis (Giuliano et al., 2012; Mesholam-Gately, Giuliano, Goff, Faraone, & Seidman, 2009). However, follow-up studies of CHR cohorts have shown inconsistent evidence for a decline (Bora & Murray, 2014): some have reported a decline in visual memory, processing speed (Wood et al., 2007) and verbal fluency (Lee et al., 2014), whereas others have observed stable cognitive deficits over time (Allott et al., 2019; Metzler et al., 2015). An improved understanding of longer-term cognitive changes in CHR populations, and particularly over illness onset for those who transition to psychosis (CHR-T), may provide insights for clinical research and inform early interventions targeting cognitive decline (Catalan et al., 2021).

To date, only one systematic review and meta-analysis has examined longitudinal changes in neurocognitive function of CHR individuals (Bora & Murray, 2014). Results indicated a general improvement in performance over time (i.e. stability of deficits), which did not significantly differ between CHR individuals and HCs with the exception of the verbal fluency domain. Here, the magnitude of improvement was significantly more pronounced in HC than in the CHR group. The main limitation of the meta-analysis was the small number of included studies, which meant that individual-task analysis was not always feasible. Instead, task performance was combined and analysed as global or domain-level cognition scores (Bora & Murray, 2014). Since this meta-analysis, several large cohort studies have published results on longitudinal neurocognition in CHR samples and over longer follow-up times, including the North American Prodrome Longitudinal Study (NAPLS-2) (Addington et al., 2019; Velikonja et al., 2021) and the Personal Assessment and Crisis Evaluation (PACE) Clinic (Allott et al., 2019). Given the increase in published studies examining longitudinal neurocognitive performance in CHR samples, an updated review is required.

The aim of the present study was to meta-analytically examine changes in neurocognitive functioning in specific tasks over two assessments among (1) CHR compared to HCs, as well as (2) CHR-T compared to CHR-NT individuals. In the current paper, we sought to address some of the limitations in the design of the earlier meta-analysis. First, we aimed to conduct analyses of performance in individual tasks, which may be a more effective approach for identifying longitudinal changes in specific cognitive processes, some of which may be differentially impaired (Brewer et al., 2006; Szöke et al., 2008). Second, we extended the analysis to examine whether changes in specific neurocognition were associated with transition to psychosis among CHR, which may help to characterise CHR-T individuals (Fusar-Poli et al., 2020). Third, we have applied a robust method for calculating effect sizes from data collected at multiple time points recommended by Morris (2008), who has comprehensively assessed the precision and stability of effect sizes from repeated measures designs.

## Methods

### Selection procedure

The systematic review protocol was registered on PROSPERO (CRD42020207568) and followed the Preferred Reporting Items for Systematic Reviews and Meta-Analyses (PRISMA) guidelines (Moher, Liberati, Tetzlaff, & Altman, 2009). Three independent authors (E.H., S.S., C.S.) carried out systematic literature searches in Medline, Embase and PsycINFO databases until 31 December 2021. Identified articles were screened first by title and abstract for possible inclusion. Full text of relevant papers was then reviewed for eligibility. A manual search of the reference lists of included articles was also conducted.

### Search strategy and eligibility criteria

Literature searches were implemented using the following key terms: (‘at risk mental state’ OR ‘ultra high risk’ OR ‘UHR’ OR ‘clinical high risk’ OR ‘psychosis risk’ OR ‘prodrome’ OR ‘psychosis’ OR ‘basic symptoms’) AND (‘neurocognit*’ OR ‘cognit*’ OR ‘neuropsych*’) AND (‘retest’ OR ‘longitudinal’ OR ‘chang*’ OR ‘follow-up’ OR ‘course’).

Studies were included if they (1) were original research articles published in English; (2) included individuals who met CHR criteria, as defined by any validated scale including the Comprehensive Assessment of At-Risk Mental States (CAARMS) (Yung et al., 2005) and Structured Interview for Psychosis-risk Syndromes (SIPS) (McGlashan, Walsh, & Woods, 2010); (3) included a comparison group of HCs or provided data separately for CHR-T and CHR-NT groups; (4) reported raw neurocognitive test scores from two assessments and (5) administered the same cognitive test at both assessments. Studies were excluded if they: (1) were unpublished studies, reviews, conference abstracts or case reports; (2) had overlapping samples on the same cognitive measure; (3) only examined cognitive performance in FEP, schizophrenia or bipolar disorder samples (no CHR sample); (4) included intervention therapies to improve cognition between assessments and (5) reported only composite cognition or standardised *z*-scores in the original article and could not provide the raw data upon request. For example, when studies only reported composite or standardised scores, corresponding authors were contacted by email to obtain the raw group data on individual task performance. For overlapping samples, the study with the largest sample size was chosen.

### Data extraction and risk of bias

Three researchers independently extracted data from included studies using a structured coding form (E.H., C.S., S.S.). Sample characteristics (e.g. number of subjects at first and second assessment, age at baseline, follow-up months) and details of neurocognitive measures [e.g. task used, domain, means and standard deviations (s.d.s) of results at both assessments] were extracted for CHR, HC, CHR-T and CHR-NT groups. The means and s.d.s of subgroups (i.e. CHR-T and CHR-NT) were pooled together using Equations 23.2 and 23.3 (Borenstein, Hedges, Higgins, & Rothstein, 2009) to calculate performance for an overall group (i.e. CHR), if it was not reported. Data extraction forms were compared to verify accuracy. Any inconsistencies were resolved under supervision of senior researchers (M.K., H.D.). Study risk of bias was assessed using a modified version of the Newcastle–Ottawa Scale (NOS) for cohort studies, which rates study quality from 0 to 8 stars across three categories: selection, comparability and exposure/outcome (Wells et al., 2011) (online Supplementary Table S1). Although there is no threshold for determining ‘good’ quality studies, accumulating stars index reduced risk of bias. This tool has been validated for longitudinal observational studies and has been used in previous meta-analyses of CHR samples (Catalan et al., 2021; Fusar-Poli et al., 2012; Salazar de Pablo, Catalan, & Fusar-Poli, 2020).

### Outcome measures

Across studies, neurocognitive data were grouped by task and group comparisons (CHR *v.* HC; CHR-T *v.* CHR-NT). Each task was separately meta-analysed. To ensure analyses were sufficiently powered, tasks with less than three independent studies were excluded from the meta-analyses. Individual tasks that were analysed included Trail Making Test A (TMT-A) and B (TMT-B), Brief Assessment of Cognition Scale (BACS) symbol coding, semantic fluency, letter fluency, Continuous Performance Task – Identical Pairs (CPT-IP), Rey Auditory Verbal Learning Test (RAVLT) immediate recall, California Verbal Learning Test (CVLT) immediate recall, Wechsler Adult Intelligence Scale (WAIS) block design and digit span. For consistency of interpretations, task outcome measures were categorised into neurocognitive domains based on the Measurement and Treatment Research to Improve Cognition in Schizophrenia (MATRICS) criteria (Kern et al., 2008; Nuechterlein et al., 2008) and in line with two published meta-analyses examining baseline cognition in CHR populations (Catalan et al., 2021; Fusar-Poli et al., 2012). These included: (1) processing speed, (2) attention/vigilance, (3) verbal learning and memory, (4) visuospatial ability, (5) executive functioning and (5) working memory (see online Supplementary Methods 1 for individual tasks involved).

### Statistical analyses

Methods used by researchers to calculate effect sizes from repeated measures designs have been examined in terms of precision, robustness and bias. Morris (2008) proposed an optimal methodology which calculates the effect size using the pre- and post-condition means, s.d.s and sample sizes of two independent groups. Therefore, for each study, we calculated the Hedges' *g* effect size (which is the Cohen's effect size corrected for small sample bias; Lakens, 2013) and its variance from Equations 8 and 25 provided by Morris (2008). If participants were lost to follow-up, sample sizes at the second assessment were used in the meta-analyses. The effect size variance requires an estimation of the correlation coefficient, rho, between first and second neurocognitive measures. Although rho is not usually reported in publications, it can be calculated from study data if the mean (and s.d.) pre, post and change values are reported. These data were provided by Allott et al. (2019) and the mean weighted rho across neurocognitive tasks was determined at 0.64 [95% confidence interval (CI) 0.58–0.70]. Therefore, rho was set to 0.65 for each meta-analysis but was adjusted from 0.65 to 0.58 and 0.70 in sensitivity analyses, consistent with the CIs from Allott et al. (2019), to examine the strength of results. To enable direct comparisons of effect sizes, we used the same methodological approach across all the neurocognitive tasks. However, this methodology for calculating effect size estimates assumes homogeneity of variance between the comparison groups (Morris, 2008). We used Bartlett's (1937) test to assess the assumption of equal variances. For studies where this assumption of homogeneity did not hold, we conducted a second sensitivity analysis to verify our findings. In the sensitivity analysis, Hedges' *g* study effect sizes were recalculated from equations provided by Morris and DeShon (2002) which do not rely on the assumption of equal variances (Equation 6 and the corresponding sampling variance in Table 2).

For each neurocognitive task, study effect sizes were combined using a random-effects inverse-weighted variance model (DerSimonian & Laird, 1986) for (1) CHR participants *v.* HCs and (2) CHR-NT *v.* CHR-T participants. Meta-analyses were conducted in Microsoft Excel using standard meta-analytical equations taken from the Major Depressive Disorder Neuroimaging Database (Kempton et al., 2011), which are identical to the METAN command (Llamas-Velasco, Contador, Villarejo-Galende, Lora-Pablos, & Bermejo-Pareja, 2015) in STATA (StataCorp, 2017). In terms of validation, previous meta-analyses have used this method in parallel with STATA and produced the same results (Bromis, Calem, Reinders, Williams, & Kempton, 2018; Kempton et al., 2011). In the meta-analyses, where changes in neurocognition from baseline to follow-up significantly differed between groups, estimated mean scores were plotted to visualise these changes in performance. To note, as our analyses examine change over time, we are not able to comment on statistically significant group differences at individual time points. Between-study heterogeneity was estimated using the Cochran *Q* test (χ^2^ and *p* value) and the degree of heterogeneity was measured by the *I*^2^ statistic. *I*^2^ values above 75% indicate high heterogeneity (Higgins, Thompson, Deeks, & Altman, 2003). Potential effect size moderators can be explored using meta-regression when at least 10 studies are available (Sharp, 1998). Publication bias was assessed using the Egger's test (Egger, Smith, Schneider, & Minder, 1997) when at least six studies were included to ensure the test was adequately powered (Sutton, Duval, Tweedie, Abrams, & Jones, 2000). Tests were two-sided and statistical significance was set at *p* < 0.05.

## Results

### Study characteristics

Of 9804 unique articles that were identified in the literature search, 76 full-text articles were assessed for possible inclusion (see online Supplementary Fig. S1 for the study selection procedure). Seven authors were successfully contacted to provide additional neurocognitive data required for the meta-analysis (Addington et al., 2019; Allott et al., 2019; Barbato et al., 2013; Fujioka et al., 2020; Lam et al., 2018; Lee et al., 2014; Liu et al., 2015). Thirteen studies met inclusion criteria for the meta-analyses (Addington et al., 2019; Allott et al., 2019; Barbato et al., 2013; Becker et al., 2010; Fujioka et al., 2020; Jahshan et al., 2010; Lam et al., 2018; Lee et al., 2014; Liu et al., 2015; Metzler et al., 2015; Shin et al., 2016; Wood et al., 2007; Woodberry et al., 2013) (see online Supplementary Table S2 for characteristics of the study database). Although there were overlapping samples for certain tasks reported by (1) Allott et al. (2019) and Wood et al. (2007), and (2) Lee et al. (2014) and Shin et al. (2016), only the latter of each pair included a HC group. Therefore, these two studies (Shin et al., 2016; Wood et al., 2007) were included in CHR *v.* HC meta-analyses. Follow-up time of studies ranged from 6 months to 13.1 years (online Supplementary Table S2).

### Longitudinal neurocognitive functioning in CHR compared to HC individuals

**Fig. 1.**
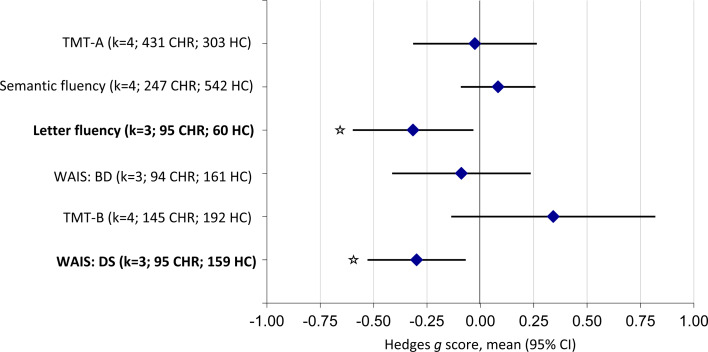
Neurocognitive task-level functioning in CHR individuals compared to HC individuals. A negative effect size demonstrated an improvement in the HC compared to the CHR group. However, this is reversed for TMTs as higher scores indicate poorer performance on these tasks. Tasks highlighted in bold indicate significant results (*p* < 0.05).

Eight studies were included in the CHR *v.* HC meta-analyses, comprising a total of 794 CHR and 787 HC individuals. Changes in neurocognitive performance significantly differed between CHR and HC individuals on letter fluency tests (*g* = −0.32; 95% CI −0.60 to −0.03; *p* = 0.029) and WAIS digit span (*g* = −0.30; 95% CI −0.53 to −0.07; *p* = 0.011) (online Supplementary Figs S2 and S4). For letter fluency, HCs improved significantly more than the CHR group (online Supplementary Fig. S3). For WAIS digit span, results indicated that there were little differences in performance at baseline, but HCs improved over time and CHR individuals did not (online Supplementary Fig. S5). There were no differences in TMT-A, semantic fluency, WAIS block design or TMT-B tasks (Fig. 1; Table 1), indicating that there was no significantly different improvement between CHR and HC groups.

**Table 1.** Neurocognitive task-level functioning of individuals at CHR compared with HC individuals

Task	Studies	Number of participants		Mean baseline score^a^		Mean change score at follow-up^a^		Effect size				Heterogeneity		
		CHR	HC	CHR	HC	CHR	HC	Hedges' *g*	95% CI	*z*	*p* value	*Q*	*I*^2^	*p* value
TMT-A	4	431	303	30.18	26.45	−3.55	−3.36	−0.03	−0.32 to 0.26	0.17	0.862	8.53	64.82	0.036*
Semantic fluency	4	247	542	33.65	39.45	0.99	0.57	0.08	−0.09 to 0.26	0.93	0.352	4.06	26.04	0.255
**Letter fluency**	3	95	60	29.19	30.23	0.48	3.40	−0.32	−0.60 to −0.03	2.19	0.029*	0.37	0.00	0.834
WAIS-R/III block design	3	94	161	21.97	24.37	2.04	1.70	−0.09	−0.41 to 0.24	0.54	0.592	3.39	40.99	0.184
TMT-B	4	145	192	71.10	60.98	−0.52	−8.53	0.34	−0.14 to 0.82	1.40	0.162	14.46	79.26	0.002*
**WAIS-R/III digit span**	3	95	159	11.18	11.13	−0.07	1.00	−0.30	−0.53 to −0.07	2.54	0.011*	1.44	0.00	0.487

Tasks highlighted in bold indicate significant results (*p* < 0.05).

a Estimated as the non-weighted mean performance from included studies.

*p < 0.05.

### Longitudinal neurocognitive functioning in CHR-T compared to CHR-NT individuals

**Fig. 2.**
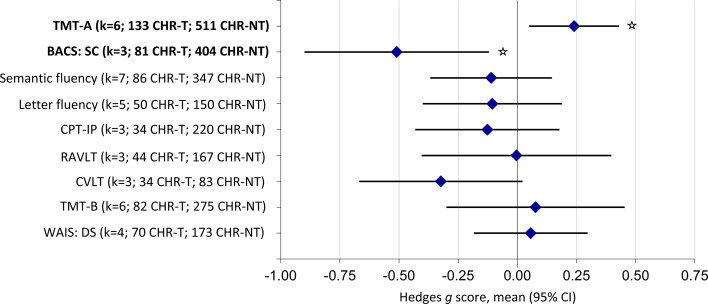
Neurocognitive task-level functioning in CHR individuals who developed psychosis (CHR-T) compared to those who did not develop psychosis (CHR-NT). A negative effect size demonstrated an improvement in the CHR-NT compared to the CHR-T group. However, this is reversed for TMTs as higher scores indicate poorer performance on these tasks. Tasks highlighted in bold indicate significant results (*p* < 0.05).

Eleven studies were included in the CHR-T *v.* CHR-NT meta-analyses, consisting of 227 CHR-T and 806 CHR-NT individuals. Changes in neurocognitive performance differed between CHR-T and CHR-NT individuals in TMT-A task (*g* = 0.24; 95% CI 0.05–0.43; *p* = 0.014) and BACS symbol coding subtest (*g* = −0.51; 95% CI −0.89 to −0.12; *p* = 0.011) (online Supplementary Figs S6 and S8). For TMT-A, CHR-NT improved significantly more than the CHR-T group (online Supplementary Fig. S7). For BACS symbol coding, CHR-T had higher scores than CHR-NT at baseline. However, CHR-T performance had worsened at follow-up, where CHR-NT had improved over time (online Supplementary Fig. S9). There were no significant differences in semantic or letter fluency, CPT-IP, RAVLT, CVLT, TMT-B or WAIS digit span tests (Fig. 2; Table 2). Results indicated that CHR-T and CHR-NT group performance on these tasks were both unchanged at follow-up, or had improved at a similar rate over time.

**Table 2.** Neurocognitive task-level functioning of individuals at clinical high-risk for psychosis who did (CHR-T) and did not (CHR-NT) transition to psychosis

Task	Studies	Number of participants		Mean baseline score^a^		Mean change score at follow-up^a^		Effect size				Heterogeneity			Publication bias
		CHR-T	CHR-NT	CHR-T	CHR-NT	CHR-T	CHR-NT	Hedges' *g*	95% CI	*z*	*p* value	*Q*	*I*^2^	*p* value	*p* value
**TMT-A**	6	133	511	32.07	29.36	−0.45	−3.99	0.24	0.05 to 0.43	2.46	0.014*	5.82	14.11	0.324	0.11
**BACS symbol coding**	3	81	404	65.47	61.52	−4.33	4.45	−0.51	−0.89 to −0.12	2.56	0.011*	4.16	51.90	0.125	–
Semantic fluency	7	86	347	29.02	32.43	−1.08	0.70	−0.16	−0.44 to 0.11	1.15	0.249	10.22	41.31	0.116	0.77
Letter fluency	5	50	150	23.63	25.45	−0.96	1.74	−0.14	−0.42 to 0.15	0.94	0.347	0.97	0.00	0.914	–
CPT-IP	3	34	220	1.40	3.30	0.34	−0.10	−0.13	−0.43 to 0.18	0.81	0.416	0.71	0.00	0.702	–
RAVLT	3	44	167	41.45	46.08	1.58	0.86	0.00	−0.40 to 0.40	0.02	0.986	3.65	45.18	0.161	–
CVLT/-II	3	34	83	36.50	39.37	1.04	2.64	−0.32	−0.67 to 0.02	1.84	0.066	0.82	0.00	0.662	–
TMT-B	6	82	275	75.15	68.10	−3.55	−5.58	0.08	−0.30 to 0.45	0.40	0.689	14.81	66.24	0.011*	0.40
WAIS/-R/II digit span	4	70	173	11.83	13.67	0.30	0.15	0.06	−0.18 to 0.30	0.46	0.643	1.68	0.00	0.641	–

Tasks highlighted in bold indicate significant results (*p* < 0.05).

a Estimated as the non-weighted mean performance from included studies.

*p < 0.05.

### Heterogeneity, study quality and publication bias

Heterogeneity across the studies was small to high (Tables 1 and 2). Potential effect size moderators could not be explored due to insufficient power to perform meta-regressions. Where publication bias could be assessed, we reported no significant evidence of bias (all *p* > 0.05) (Table 2). In terms of study risk of bias, NOS scores ranged from five to seven (mean = 5.88; median = 6.00) in the CHR *v.* HC meta-analysis and from two to seven (mean = 5.45; median = 6.00) in the CHR-T *v.* CHR-NT meta-analysis (online Supplementary Table S2).

### Sensitivity analysis

By increasing rho (the correlation between baseline and follow-up neurocognitive measures) to 0.70, no change in significant results was observed. We did, however, detect an additional significant difference in longitudinal performance of CVLT among CHR-T and CHR-NT groups (*g* = −0.32; 95% CI −0.64 to −0.004; *p* = 0.047), where the CHR-NT had improved more than CHR-T. When rho was modified to 0.58, there was no change in significant results compared to rho at 0.65.

We conducted a second sensitivity analysis recalculating study effect sizes where we could not assume homogeneity of variance of comparison groups. In the sensitivity analysis, significant differences between CHR and HC in longitudinal performance of letter fluency (*g* = −0.33; 95% CI −0.62 to −0.04; *p* = 0.046) and WAIS digit span (*g* = −0.30; 95% CI −0.53 to −0.07; *p* = 0.011) remained. In keeping with our earlier findings, we also reported differences in longitudinal performance of TMT-A (*g* = 0.30; 95% CI 0.06 to 0.55; *p* = 0.016) and BACS symbol coding (*g* = −0.51; 95% CI −0.90 to −0.11; *p* = 0.012) among CHR-T and CHR-NT individuals. Therefore, the results of the second sensitivity analysis supported those of our main analysis.

## Discussion

In this systematic review and meta-analysis, we first observed that longitudinal improvements in verbal fluency and digit span task performance were significantly more pronounced in HC compared to CHR individuals. Our second main finding was that performance over time in TMT-A and symbol coding tasks significantly differed between CHR-T and CHR-NT individuals. Whilst CHR-NT improved in performance on both tasks, CHR-T improved to a lesser degree in TMT-A and had worsened performance in symbol coding at follow-up. To our knowledge, this is the first comprehensive meta-analysis of longitudinal neurocognitive task performance in CHR-T and CHR-NT samples.

Our meta-analysis of longitudinal neurocognition in 697 CHR and 761 HC individuals demonstrated that performance in both groups generally improved between baseline and follow-up assessments. This may reflect the magnitude of practice effects, particularly for meta-analyses that included studies with shorter follow-up intervals (Calamia, Markon, & Tranel, 2012). We did, however, detect small effect size differences in longitudinal performance of digit span and letter fluency tasks, where improvements at follow-up were significantly more pronounced in HCs. An earlier meta-analysis of longitudinal cognition reported the same findings for letter fluency but did not have enough studies to analyse digit span performance in CHR (Bora & Murray, 2014). However, deficits in digit span are well-established in FEP (Mesholam-Gately et al., 2009) and schizophrenia patients (Fatouros-Bergman, Cervenka, Flyckt, Edman, & Farde, 2014). Furthermore, Bora and Murray (2014) did report that improvements over follow-up in the working memory domain, which comprises digit span performance, were significantly greater in HC than FEP. Findings are also in line with birth cohort studies that report developmental lags in cognitive performance from childhood at age 8 years among adults with psychotic disorder compared to HC individuals (Mollon, David, Zammit, Lewis, & Reichenberg, 2018). Developmental lags in cognitive functioning have also been identified between ages 9 and 16 years among children at-risk who present with a triad of antecedent markers of schizophrenia compared to typically developing children (Dickson et al., 2018). Our results showed reduced cognitive improvement of CHR individuals between assessments, which may reflect underlying structural and functional brain abnormalities in the prefrontal and anterior cingulate cortex of CHR and FEP individuals; key regions of working memory and verbal fluency function (Fusar-Poli et al., 2007, 2011). Still, our results should be interpreted cautiously as we are limited by heterogeneity attributable to both the CHR phenotype and to primary studies, such as short follow-up times (up to 2 years).

Prior research has suggested that any potential decline in neurocognition may be specific to individuals who transition to psychosis (Bora & Murray, 2014). As stated earlier, only one meta-analysis has examined the course of neurocognition in CHR across two assessments, but there was insufficient data to conduct task analysis for CHR-T and CHR-NT groups (Bora & Murray, 2014). Of nine tasks analysed in the present review, we observed small to moderate effect sizes differences in longitudinal processing speed, indexed by performance on both TMT-A (*g* = 0.24) and symbol coding tasks (*g* = −0.51). Improvements in TMT-A were significantly more pronounced among CHR-NT than CHR-T individuals. For the symbol coding task, performance was in fact higher in the CHR-T group at baseline but there was evidence of worsening performance at the follow-up assessment, whereas the CHR-NT group had improved. Interestingly, processing speed, and specifically symbol coding, has been recognised as the largest deficit in schizophrenia (Dickinson, Ramsey, & Gold, 2007), as well as in CHR samples (Seidman et al., 2010), relative to other common neurocognitive measures. Our results may indicate that some decline or lag in processing speed performance may occur later during the prodromal phase in those who develop psychosis (Seidman et al., 2010). This is of importance given the known relationship between poorer performance on trail making and symbol coding tasks and poorer social and role functioning among CHR individuals (Carrión et al., 2011) and highlights the need to develop interventions to address these impairments prior to the onset of psychosis. Although few randomised controlled trials (RCTs) have examined the effectiveness of cognitive remediation therapies on neurocognition and functioning in CHR groups, some do provide evidence that cognitive remediation may improve performance in select cognitive domains, such as processing speed and verbal memory (Choi et al., 2017; Loewy et al., 2016), and social functioning (Friedman-Yakoobian, Parrish, Eack, & Keshavan, 2022; Piskulic, Barbato, Liu, & Addington, 2015). Of interest, in a double-blind RCT directly targeting processing speed deficits, CHR participants who underwent processing speed training had significantly improved scores on WAIS-III symbol coding task as well as enhanced social adjustment at follow-up compared to an active control group (Choi et al., 2017).

To our knowledge, this is the largest comprehensive meta-analysis characterising longitudinal neurocognitive functioning in CHR individuals to date. We have extended previous research by Bora and Murray (2014) to compare changes in specific task performance of CHR-T and CHR-NT individuals. An additional strength of our review is that we applied a robust analytic approach to calculate effect sizes from repeated measures designs (Morris, 2008) and our results did not change during sensitivity analyses. However, limitations of the current paper must also be noted. There were several tasks that could not be meta-analytically examined due to an insufficient number of included studies and our approach to analyse the data at the task level. Though heterogeneity was typically low, considerable heterogeneity was observed for TMT-B in the meta-analyses. However, due to limited studies, we could not perform meta-regression analyses to investigate heterogeneity, exploring potential moderator variables, such as changes in symptoms, medication use or length of follow-up (which may reflect practice effects). Lastly, although we were able to examine changes in neurocognition of CHR, our meta-analyses consisted of data collected from two assessments. As a result, the interpretation of our findings is limited. Of 13 articles included in the meta-analyses, two studies examined neurocognition at more than two assessments but had small sample sizes for the transition group at follow-up (Lam et al., 2018; Lee et al., 2014). Therefore, we have limited insight into the nonlinear trajectories of neurocognition in CHR and over psychosis onset in CHR-T. Future research collecting repeated data at multiple time points in larger CHR cohorts is warranted.

To conclude, the current meta-analysis suggests that, despite general improvements in neurocognition among CHR, there are some differences in task performance over 2 years in CHR compared to HC as well as CHR-T relative to CHR-NT. These longitudinal differences were observed in processing speed and working memory domains. Taken together, these results suggest that tasks related to processing speed and working memory may be key targets for interventions aimed at improving neurocognitive deficits in clinical high-risk populations.

## Supplementary material

For supplementary material accompanying this paper visit https://doi.org/10.1017/S0033291722001830. click here to view supplementary material
