# Supplementary material for: Meta-analysis of longitudinal neurocognitive performance in people at clinical high-risk for psychosis
Source: Psychol Med. 2022 Jul 13;52(11):2009–16. doi: 10.1017/S0033291722001830 (PMC9386433; doi:10.1017/S0033291722001830)
Supplement: Supplementary file 1 [file S0033291722001830sup001.docx]

# Supplementary Materials

**eMethods 1.** Neurocognitive domains and individual tasks included in the current meta-analysis

**eTable 1.** Risk of bias (quality) assessment using modified Newcastle-Ottawa Scale for cohort studies

**eTable 2.** Baseline characteristics of included studies

**eFigure 1.** PRISMA flow chart outlining the study selection procedure

**eFigure 2.** Forest plot representing the meta-analysis of changes in letter fluency performance for individuals at clinical high-risk for psychosis (CHR) compared with healthy control (HC) individuals

**eFigure 3.** Graph displaying mean performance on letter fluency tasks of included studies (n = 3) at baseline and follow-up for individuals at clinical high-risk for psychosis (CHR) compared with healthy control (HC) individuals

**eFigure 4.** Forest plot for representing the meta-analysis of changes in WAIS digit span performance for individuals at clinical high-risk for psychosis (CHR) compared with healthy control (HC) individuals

**eFigure 5.** Graph displaying mean performance on WAIS digit span of included studies (n = 3) at baseline and follow-up for individuals at clinical high-risk for psychosis (CHR) compared with healthy control (HC) individuals

**eFigure 6.** Forest plot for representing the meta-analysis of changes in TMT-A performance for individuals at clinical high-risk for psychosis who did (CHR-T) and did not (CHR-NT) transition to psychosis

**eFigure 7.** Graph displaying mean performance on TMT-A tasks of included studies (n = 6) at baseline and follow-up for individuals at clinical high-risk for psychosis who did (CHR-T) and did not (CHR-NT) transition to psychosis

**eFigure 8.** Forest plot for representing the meta-analysis of changes in BACS symbol coding performance for individuals at clinical high-risk for psychosis who did (CHR-T) and did not (CHR-NT) transition to psychosis

**eFigure 9.** Graph displaying mean performance on BACS symbol coding tasks of included studies (n = 3) at baseline and follow-up for individuals at clinical high-risk for psychosis who did (CHR-T) and did not (CHR-NT) transition to psychosis.

**eMethods 1:** Neurocognitive domains and individual tasks included in the current meta-analysis

| Neurocognitive Domains | Tasks |
| --- | --- |
| Processing speed | - Trail Making Test A (TMT-A)^1,2^ - Brief Assessment of Cognition Scale (BACS) symbol coding^3,4^ - Semantic fluency^4,5,6,7,8^ - Letter fluency^4,5,6,8^ |
| Attention/vigilance | - Continuous Performance Task - Identical Pairs I/II (CPT-IP)^9,10^ |
| Verbal learning and memory | - Rey Auditory Verbal Learning Test (RAVLT)^11,12^ - California Verbal Learning Test I/II (CVLT)^13,14,15^ |
| Visuospatial ability | - Wechsler Adult Intelligence Scale R/III (WAIS) block design^16,17^ |
| Executive functioning | - Trail Making Test B^1,2^, Delis-Kaplan Executive Function System Trail Making Condition 4 (TMT-B)^18^ |
| Working memory | - WAIS I/R/III digit span^16,19,20^ |

**eTable 1.** Risk of bias (quality) assessment using modified Newcastle-Ottawa Scale for cohort studies

| **Newcastle-Ottawa Scale Criteria** | **Maximum Score** |
| --- | --- |
| *Selection* | |
| Representativeness of the exposed cohort (e.g., random sample selected group) | 1 |
| Selection of the non-exposed cohort | 1 |
| Ascertainment of exposure (i.e., secure record or structured interview) | 1 |
| *Comparability* | |
| Comparability of cohorts on the basis of the design or analysis (i.e., groups are matched or adjustment for confounding factors) | 2 |
| *Outcome* | |
| Assessment of outcome (were robust tools used?) | 1 |
| Was follow-up long enough for outcomes to occur? | 1 |
| Loss to follow-up is low (<30%) and same in exposed and non-exposed? | 1 |

**eTable 2.** Baseline characteristics of included studies

| **Study** | **Sample** | **Sample size, N** | | **Age, M(SD)** | | **FUP (mo)** | **NOS** | **Tasks analysed** |
| --- | --- | --- | --- | --- | --- | --- | --- | --- |
| **CHR versus HC meta-analysis** | | *HC* | *CHR* | *HC* | *CHR* |  | | |
| Wood et al. (2007) | Personal Assessment and Crisis Evaluation Clinic, Australia | 17 | 16 | 19.7 (2.4) | NR | 12 | 6 | TMT-A, letter fluency, WAIS-R block design, TMT-B, WAIS-R digit span |
| Becker et al. (2010) | Academic Medical Centre, The Netherlands | 17 | 41 | 19.4 (3.8) | NR | 18 | 5 | Semantic fluency, letter fluency |
| Jahsan et al. (2010) | Cognitive Assessment and Risk Evaluation, USA | 29 | 46 | 19.0 (5.2) | 18.7 (4.2) | 6-36 | 5 | WAIS-III block design |
| Woodberry et al. (2013) | FACT Study, USA | 32 | 53 | 16.3 (2.6) | 16.0 (2.4) | 12 | 6 | D-KEFS trail making 4 |
| Liu et al. (2015) | SOPRES Study, Taiwan | 137 | 53 | 21.4 (4.1) | 21.4 (3.9) | 12 | 7 | TMT-A, semantic fluency, WAIS-III block design, TMT-B, WAIS-III digit span |
| Shin et al. (2016) | Seoul Youth Clinic, South Korea | 28 | 47 | 27.0 (6.0) | 19.3 (3.3) | 24 | 6 | TMT-A, semantic fluency, letter fluency, TMT-B, WAIS-K digit span |
| Lam et al. (2018) | Longitudinal Youth At-Risk Study, Singapore | 384 | 173 | 21.7 (3.3) | 21.3 (3.5) | 24 | 7 | Semantic fluency |
| Addington et al. (2019) | North American Prodrome Longitudinal Study 2, USA | 143 | 366 | NR | NR | 24 | 5 | TMT-A |
| **CHR-T versus CHR-NT meta-analysis** | | *CHR-NT* | *CHR-T* | *CHR-NT* | *CHR-T* |  | | |
| Wood et al. (2007) | Personal Assessment and Crisis Evaluation Clinic, Australia | 9 | 7 | 21.0 (3.1) | 17.3 (2.8) | 12 | 6 | Letter fluency |
| Becker et al. (2010) | Academic Medical Centre, The Netherlands | 24 | 17 | 19.2 (2.8) | 20.8 (4.4) | 18 | 6 | Semantic fluency, letter fluency, CVLT |
| Barbato et al. (2013) | PREDICT Study, USA | 72 | 9 | 19.8 (4.7) | NR | 6 | 2 | TMT-A, semantic fluency, CPT-IP, RAVLT, TMT-B |
| Woodberry et al. (2013) | FACT Study, USA | 43 | 10 | 16.0 (2.4) | 16.1 (2.1) | 12 | 6 | CPT-IP-II, CVLT-II, D-KEFS trail making 4 |
| Lee et al. (2014) | Seoul Youth Clinic, South Korea | 61 | 14 | 19.8 (3.5) | 20.6 (4.9) | 24 | 7 | TMT-A, semantic fluency, letter fluency, K-CVLT, TMT-B, WAIS-K digit span |
| Liu et al. (2015) | SOPRES Study, Taiwan | 35 | 18 | 21.4 (3.9) | | 12 | 5 | TMT-A, semantic fluency, TMT-B, WAIS-III digit span |
| Metzler et al. (2015) | ZInEP, Switzerland | 60 | 12 | 20.8 (6.1) | 19.1 (4.8) | 12 | 6 | TMT-A, semantic fluency, letter fluency, RAVLT, TMT-B, WAIS digit span |
| Lam et al. (2018) | Longitudinal Youth At-Risk Study, Singapore | 156 | 17 | 21.4 (3.6) | 20.4 (3.2) | 24 | 7 | BACS symbol coding, semantic fluency, CPT-IP |
| Addington et al. (2019) | North American Prodrome Longitudinal Study 2, USA | 278 | 88 | 18.8 (4.4) | NR | 24 | 5 | TMT-A, BACS symbol coding |
| Allott et al. (2019) | Personal Assessment and Crisis Evaluation Clinic 1994 - 2000, Australia | 49 | 31 | 20.2 (3.2) | | 87-157 | 7 | TMT-A, RAVLT, TMT-B, WAIS-R digit span |
| Fujioka et al. (2020) | IN-STEP Study, Japan | 21 | 3 | 20.4 (3.7) | | 6-37 | 3 | BACS-J symbol coding, semantic fluency, letter fluency |

HC Healthy controls; CHR Clinical high-risk; FUP follow-up period; NOS Newcastle-Ottawa Scale; TMT Trail making test; WAIS Weschler Adult Intelligence Scale; D-KEFS Delis-Kaplan Executive Function System; CVLT California Verbal Learning Test; CPT-IP Continuous Performance Test – Identical Pairs; RAVLT Rey Auditory Verbal Learning Test; BACS Brief Assessment of Cognition Scale; NR Not reported

**eFigure 1:** PRISMA flow chart outlining the study selection procedure

Records excluded
(n = 9728)

Records screened
(n = 9804)

Records after duplicates removed
(n = 9804)

Full-text articles excluded, with reasons
(n = 63)

- CHR criteria not met (n = 10)

- No measure of cognition (n = 10)

- Cognition measured at one time point (n = 33)

- Participants taking part in a treatment trial (n = 1)

- Overlapping sample (n = 1)

- Insufficient data for meta-analysis (n = 4)

- Author could not provide data to calculate effect size (n = 4)

Studies included in quantitative synthesis (meta-analysis)
(n = 13)

Full-text articles assessed for eligibility
(n = 76)

Additional records identified through other sources
(n = 12)

Records identified through database searching
(n = 14 919)

## Included

## Identification

## Eligibility

## Screening

**eFigure 2.** Forest plot representing the meta-analysis of changes in letter fluency performance for individuals at clinical high-risk for psychosis (CHR) compared with healthy control (HC) individuals

**eFigure 3.** Graph displaying mean performance on letter fluency tasks of included studies (n = 3) at baseline and follow-up for individuals at clinical high-risk for psychosis (CHR) compared with healthy control (HC) individuals

**eFigure 4.** Forest plot for representing the meta-analysis of changes in WAIS digit span performance for individuals at clinical high-risk for psychosis (CHR) compared with healthy control (HC) individuals

**eFigure 5.** Graph displaying mean performance on WAIS digit span of included studies (n = 3) at baseline and follow-up for individuals at clinical high-risk for psychosis (CHR) compared with healthy control (HC) individuals.

**eFigure 6.** Forest plot for representing the meta-analysis of changes in TMT-A performance for individuals at clinical high-risk for psychosis who did (CHR-T) and did not (CHR-NT) transition to psychosis

**eFigure 7.** Graph displaying mean performance on TMT-A tasks of included studies (n = 6) at baseline and follow-up for individuals at clinical high-risk for psychosis who did (CHR-T) and did not (CHR-NT) transition to psychosis

**eFigure 8.** Forest plot for representing the meta-analysis of changes in BACS symbol coding performance for individuals at clinical high-risk for psychosis who did (CHR-T) and did not (CHR-NT) transition to psychosis

**eFigure 9.** Graph displaying mean performance on BACS symbol coding tasks of included studies (n = 3) at baseline and follow-up for individuals at clinical high-risk for psychosis who did (CHR-T) and did not (CHR-NT) transition to psychosis

# References

^1^ Reitan, R. M., & Wolfson, D. (1985). *The Halstead–Reitan Neuropsychological Test Battery: Therapy and clinical interpretation.* Tucson, AZ: Neuropsychological Press.

^2^ Adjutant General’s Office. (1944). *Army Individual Test. Manual of Directions and Scoring.* Washington DC: War Department.

^3^ Keefe, R.S., Goldberg, T.E., Harvey, P.D., Gold, J.M., Poe, M.P., & Coughenour, L. (2004). The Brief Assessment of Cognition in Schizophrenia: reliability, sensitivity, and comparison with a standard neurocognitive battery. *Schizophrenia research, 68*(2-3), 283-297.

^4^ Kaneda, Y., Sumiyoshi, T., Keefe, R., Ishimoto, Y., Numata, S., & Ohmori, T. (2007). Brief assessment of cognition in schizophrenia: validation of the Japanese version. *Psychiatry and Clinical Neurosciences, 61*(6), 602-609.

^5^ Benton, A.L.., & Hamsher, K. (1983). *Mulilingual Aphasia Examination*, Second Ed. AHA Associates, Iowa City.

^6^ Lezak, M. D. (1995). *Neuropsychological Assessment.* Oxford University Press: New York.

^7^ Keefe, R. S., Harvey, P. D., Goldberg, T. E., Gold, J. M., Walker, T. M., Kennel, C., & Hawkins, K. (2008). Norms and standardization of the Brief Assessment of Cognition in Schizophrenia (BACS). *Schizophrenia Research, 102*(1-3), 108-115.

^8^ Aschenbrenner, A., Tucha, O., & Lange, K. (2000*). Regensburger Wortflüssigkeits-Test. Handanweisung.* Hogrefe: Göttingen.

^9^ Cornblatt, B., & Keilp, J. (1994). Impaired attention genetics, and the pathophysiology of schizophrenia. *Schizophrenia Bulletin, 20*, 31–46.

^10^ Cornblatt, B. A., Risch, N. J., Faris, G., Friedman, D., & Erlenmeyer-Kimling, L. (1988). The Continuous Performance Test, Identical Pairs Version (CPT-IP): I. new findings about sustained attention in normal families. *Psychiatry Research, 26*(2), 223-238.

^11^ Rey, A. (1958). *The Clinical Examination in Psychology*. Presse Universitaire de France, Paris.

^12^ Helmstaedter, C., Lendt, M., & Lux, S. (2001). *Verbaler Lern- und Merkfähigkeitstest (VLMT).* Hogrefe: Göttingen.

^13^ Kibby, M. Y., Schmitter-Edgecombe, M., & Long, C. J. (1998). Ecological validity of neuropsychological tests: focus on the California Verbal Learning Test and the Wisconsin Card Sorting Test. *Archives of Clinical Neuropsychology, 13*, 523–534.

^14^ Kang, Y., & Kim, J. (1997). Korean-California Verbal Learning Test (K-CVLT): a normative study. Kor. *J. Clin. Psychiatry 16*, 379–396.

^15^ Delis, D.C., Kramer, J.H., Kaplan, E., & Ober, B.A. (2000). *California Verbal Learning Test, Second Ed.* Psychological Corporation, San Antonio, TX.

^16^ Wechsler, D. (1981). *Wechsler Adult Intelligence Scale - Revised Manual.* Psychological Corporation: New York.

^17^ Wechsler, D. (1997). *Wechsler Adult Intelligence Scale - Third Edition.* Psychological Corporation: San Antonio, Texas.

^18^ Delis, D.C., Kaplan, E., & Kramer, J.H. (2001). *Delis–Kaplan Executive Function System Examiner's System.* Psychological Corporation: San Antonio, TX.

^19^ Aster, M., Neubauer, A., & Horn, R. (2006). *Wechsler Intelligenztest für Erwachsene (WIE): Deutschsprachige Bearbeitung und Adaptation des WAIS-III von David Wechsler.* Harcourt Test Services: Frankfurt am Main.

^20^ Kim, Z., Lee, Y., & Lee, M. (1994). Two-and four-subtest short forms of the Korean-Wechsler Adult Intelligence Scale. *Seoul J. Psychiatry, 19*, 121–126.
